# Response to “practical implication of nitroglycerin test for diagnosing heart failure in emergency department”

**DOI:** 10.1002/clc.23721

**Published:** 2021-09-13

**Authors:** Khaoula Bel Haj Ali, Adel Sekma, Semir Nouira

**Affiliations:** ^1^ Emergency Department Fattouma Bourguiba University Hospital Monastir Tunisia; ^2^ Research Laboratory LR12SP18 University of Monastir Monastir Tunisia

To the Editor

We would like to thank the editor for giving us the opportunity to respond to the issues raised in Hiroi et al. correspondence. We would also like to express our appreciation to Hiroi and his colleagues for their interest in our article and for their knowledgeable comments. Hiroi highlights important questions. The first was about the decrease in CO after NTG test which seems to be comparable between patients with preserved ejection fraction and those without HF. We agree with Hiroi that this finding could be challenging in the distinction between heart failure with preserved ejection fraction from non‐heart failure diseases. Our results suggest that when NTG test is positive (unchanged CO) this means that heart failure is present and is most likely associated with reduced ejection fraction. This also means that when NTG test is normal (decrease of CO) additional investigations are needed including plasma B‐type natriuretic peptide and echocardiography when available. Hiroi correctly noted that cardiac output seems to decrease following NTG administration in most heart failure patients in our study. These findings are in contradiction with studies suggesting that the failing human heart is unable to use the Frank–Starling mechanism. Although previous studies on human heart preparations have yielded conflicting results with regard to preload responsiveness of failing heart, it is accepted that in congestive heart failure, the ability of the heart to utilize the Frank–Starling mechanism is preserved but attenuated.^3^ It is likely that for some patients with end stage heart failure, cardiac output would increase in heart failure patients on the descending part of the Starling curve when their preload was reduced. This preload‐CO relationship was documented in only few heart failure patients in our study. We believe that for this category of patients with obvious clinical symptoms of heart failure, the diagnostic challenge for the clinician is probably less important, and as such NTG test is probably not needed. Of course, the nitroglycerin test should not be used in patients with right ventricular infarction, hypertrophic obstructive cardiomyopathy, and severe aortic stenosis.^2^ About the timing, NTG test should be performed as early as possible. This test is very simple and can be used in a few minutes. This could be helpful when echocardiography is not immediately available in the emergency room.

## CONFLICT OF INTEREST

None.
